# Chemical modification of *Bombyx mori* silk fibers with vinyl groups for thiol-ene click chemistry

**DOI:** 10.1186/s13065-019-0630-7

**Published:** 2019-09-10

**Authors:** Xiaoning Zhang, Jianwei Liang, Zhenyu Chen, Carrie Donley, Yuling Liu, Guotao Cheng

**Affiliations:** 1 State Key Laboratory of Silkworm Genome Biology, College of Biotechnology, Southwest University, Chongqing, 400715 China; 2 Chapel Hill Analytical and Nanofabrication Laboratory, Department of Applied Physical Sciences, University of North Carolina at Chapel Hill, Chapel Hill, NC 27599-3216 USA

**Keywords:** *Bombyx mori* silk fibroin, Chemical modification, Thiol-ene click reaction

## Abstract

Natural *Bombyx mori* silk fibroin (SF) fibers were modified with 2-methacryloyloxyethyl isocyanate (MOI) first for the introduction of vinyl groups. Then, 1*H*,1*H*,2*H*,2*H*-perfluorodecanethiol was grafted onto the SF fibers via thiol-ene click chemistry using ultraviolet light. The formations of MOI-modified and PFDT-grafted SF fibers were analyzed using Fourier transform infrared spectroscopy and X-ray photoelectron spectroscopy, respectively. The morphology of samples was also revealed by a scanning electron microscope. In addition, differential scanning calorimetry results demonstrated that SF fibers did not show significant change in thermal behavior, regardless of the chemical modification. To confirm the cytotoxicity of the prepared SF fibers, MTT [3-(4,5-dimethylthiazol-2-yl)-2,5-diphenyltetrazolium bromide] assay was performed, and no toxicity was observed with PFDT-grafted SF fibers. The results also showed that PFDT-grafted SF fibers exhibited good antifouling properties when *Chlorella vulgaris* (*C. vulgaris*) was selected as a model for algal cells adhesion experiment.

## Introduction

Silkworm *Bombyx mori* can secrete silk, which consists of sericin and silk fibroin. The two paralleled fibroin fibers are held together with a layer of sericin on their surfaces [1]. Silk is a natural biopolymer and is highly appreciated for its outstanding characteristics, which majorly result from the properties and structures of silk fibroin. Unlike sericin, silk fibroin in different forms can interact with biological systems without introducing adverse immunological responses, demonstrating good biocompatibility and biodegradability [2]. Among various forms, the fibroin fibers are endowed with a combination of attractive strength, toughness, and thermal stability that surpasses many synthetic and natural fibers [3]. There is a growing interest in introducing more functionalities into silk fibroin fibers while preserving its advantageous intrinsic properties [3] in order to expand its consumption. One method, chemical modification can imbue silk fibroin fibers with new properties and functions, therefore broadening its applications in the textile and biomedical industries [4].

In 2000, Tamada et al. reported an approach to introduce a carbon double bond onto silk fibroin with 2-methacryloyloxyethyl isocyanate (MOI) [5]. Because carbon–carbon double bond can react with radicals easily, this approach allows a further modification of silk fibroin based materials, for example, polymerization with azo polymerization initiator [5]. As one example of reactions between ene functional groups and radicals, thiol-ene click reaction involves the addition of a thiyl radical across a carbon–carbon double bond in a regio- and enantio-selective manner [6]. Thiol-ene click reactions are appealing for material modification because they are rapid, largely independent of solvent, in high yield, and no need to require a radical initiator [7, 8]. To the best of our knowledge, the approach for chemical modification of SF fibers based on thiol-ene click chemistry has not been reported previously.

Our manuscript details a technique that can modify silk fibers with thiol-containing molecules based on thiol-ene click chemistry. 1*H*,1*H*,2*H*,2*H*-perfluorodecanethiol (PFDT) was used as a model molecule as fluorine atom can serve as an elemental tag. MOI, a heterofunctional monomer that combines the versatility of a vinyl double bond and a reactive isocyanate group, was used prior to the PFDT graft. Isocyanate groups can react with hydroxyl, carboxyl, and amino side groups of amino acid residues in silk fibroin, introducing vinyl groups to silk molecules and allowing them to act as reacting points. PFDT molecules were then grafted through these reacting points onto the silk by thiol-ene click reactions. Because PFDT exhibits excellent antibioadhesive properties [9], an algal cell adhesion experiment was designed to verify the antifouling property of PFDT grafted silk and furtherly prove the successful attachment of PFDT molecules onto silk fiber surface via thiol-ene click chemistry.

## Methods

### Materials

Fresh cocoons of *Bombyx mori* silkworm were obtained from College of Biotechnology, Southwest University. Sodium carbonate anhydrous (Na_2_CO_3_), calcium chloride anhydrous (CaCl_2_), and *N*,*N*-dimethylformamide (DMF) were purchased from Kelong Chemical Reagent Factory (Chengdu, China) and at AR grade. Anhydrous ethanol (CH_3_CH_2_OH, AR grade) was purchased from Chongqing Chuandong Chemical Group Co., Ltd. (Chongqing, China). 1-ethyl-3-(dimethylaminopropyl) carbodiimide hydrochloride (EDC) and *N*-hydroxysuccinimide (NHS) were purchased from Solarbio (Beijing, China). Anhydrous dimethyl sulfoxide (DMSO), dibutyltin dilaurate and hydroquinone were purchased from Aladdin Industrial Co., Ltd. (Shanghai, China). 2-Isocyanatoethyl methacrylate (MOI) was purchased from Meryer Chemical Technology Co., Ltd. (Shanghai, China). 1*H*,1*H*,2*H*,2*H*-perfluorodecanethiol (PFDT) and 1*H*,1*H*,2*H*,2*H*-perfluoro-1-decanol (FTOH) were purchased from Sigma-Aldrich (Saint-Louis, USA). Ultrapure water (resistance > 18 MΩ cm^−1^) was used in all experiments.

### Degummed silk fibroin fiber preparation

In preparation for the experiment, *Bombyx mori* silkworm cocoons were cut into dime-sized pieces. To remove the sericin, those cocoon pieces were added to 500 ml boiled sodium carbonate solution (0.047 M) for 40 min. Then, the degummed silk fibroin was removed from the boiling sodium carbonate solution and cooled by rinsing in ultrapure water three times for 20 min. Each rinsing cycle was carried out in 1 l of ultrapure water in a lab beaker, using a stir bar to circulate the volume within the beaker. After the wash, the excess water was squeezed out of the silk, and the degummed silk was then spread out on a clean piece of print paper, allowing the silk to dry in a fume hood overnight [10].

### Chemical modification by MOI monomer

Once the fibroins were dry, 0.1 g of degummed silk fibers were immersed in 12 ml of anhydrous DMSO under nitrogen protection, and 2.1 g of MOI, 2 μl of hydroquinone and 0.01 g of dibutyltin dilaurate were added to this anhydrous system. The reaction mixture was kept at 35 °C for 24 h. Chemically modified silk fibroin fibers were successively rinsed with DMSO, ethanol and large amounts of ultrapure water thoroughly to remove unreacted reagents and then lyophilized (LGJ-10, Shanghai YuMing Instrument Co., Ltd., China) for 24 h.

### Post modification via thiol-ene click reaction

To attach thiol-containing molecules to the MOI-modified SF fibers, each sample was contacted with 0.5 ml of a neat PFDT liquid. A quartz coverslip (0.5 mm thickness, Ted Pella) was placed on the sample to ensure homogeneous coverage of the organic liquid across the sample surface and was illuminated with a 40 W light-emitting diode (LED) UV lamp (UVSP1-A, ShenZhen YuXianDe Science and Technology Ltd., China) with 365 nm wavelength. Each sample was placed 20 mm below the LED UV lamp. Samples were then sonicated in ethanol to remove nonspecifically adsorbed PFDT molecules, rinsed with ultrapure water, and dried under a stream of nitrogen prior to analysis.

### Cultivation of algae

*Chlorella vulgaris* (*C. vulgaris*) was obtained from Nanjing Health Biological Technology Co. Ltd. (Nanjing, China). The algae cells were cultivated for a period of 6 days, which is when the *C. vulgaris* population reached a relatively stable phase in the culture medium before the antifouling assay. Cultures were grown at 22 ± 2 °C with 16 h of light exposes and an 8-h of dark photoperiod. Lighting was supplied by a combination of warm and cold fluorescent tubes, giving a luminance range of between 2200 and 2800 Lux. The in vivo absorption of the culture medium containing algal cells in each flask was monitored each day via a UV–Vis spectrophotometer (T6, Beijing Puxi Analytic Instrument Ltd., China) at 660 nm (the growth curve was sigmoid and is presented in Additional file 1: Figure S1).

### Silk fibroin fiber characterization

X-ray photoelectron (XP) spectroscopic characterization was carried out in an ultrahigh-vacuum system (Kratos Axis Ultra DLD, UK) with a base pressure of 5 × 10^−9^ Torr, a monochromatic Al K*α* source, and a hemispherical analyzer. Survey (1.0 eV resolution) and highresolution (0.1 eV resolution) spectra were collected at a 0° takeoff angle from surface normal, then analyzed with Kratos Vision 2.0 software. Each high-resolution spectrum was referenced to the C1s peak (284.6 eV) and fit with Voigt functions (70% Gaussian, 30% Lorentzian) after a Shirley background correction.

Scanning electron microscopy (SEM) images were acquired under the desktop SEM Phenom™ Pro (Phenom-World, Netherlands) at an accelerating voltage of 10.0 kV. Infrared spectra of samples were analyzed and recorded using Fourier transform infrared spectroscopy (FTIR, Thermo Scientific Nicolet iN10) in KBr pellets.

A differential scanning calorimeter (DSC, HSC-3, Beijing HengJiu Scientific Instrument Factory, China) utilizing N_2_ gas was used to assess the thermal behavior of the silk fibers under each processing condition. The scanning temperature was raised from room temperature to 390 °C at a heating rate of 10 °C min^−1^.

### MTT assay

The cytotoxicity assay for PFDT-grafted SF fibers was performed against Human Embryonic Kidney 293 (HEK 293) cell-lines, a kind of epithelial cells, by MTT assay as follows [11]. After sterilization with highly compressed steam for 20 min, 0.2 g of sample was immersed in 10 ml sterilized Dulbecco’s modified Eagles medium (DMEM) to equilibrate at 37 °C for 72 h. HEK 293 cells were seeded in a 96-well plate with a density of 2 × 10^3^ cells/well then incubated in 100 μl of DMEM with 10% (v/v) fetal bovine serum (FBS) for 24 h at 37 °C and 5% CO_2_. Each well was added 10 μl of leaching liquor respectively as sample group. The HEK 293 cells cultured with DMEM served as control group, and with DMEM containing 0.64% phenol served as positive group. With 24 h, 72 h, 120 h incubation, 10 μl of MTT solution (5 mg/ml in PBS) was added into each well respectively. After 4 h treatment with MTT, the medium was removed, and 200 μl of DMSO was supplemented to each well immediately to dissolve the generated formazan crystal. Later, the 96-well plate was shook in a belly dancer for 15 min to homogenize the dissolved formazan. Quantitative detection was performed on a microplate reader (Synergy H1 Hybrid Multi-Mode Reader, Gene Company Limited) at a wavelength of 570 nm. The cell viability was calculated according to equation:

$${\text{Cell viability }}\left( \% \right) = {\text{OD}}_{ 5 70} \left( {\text{sample}} \right)/{\text{OD}}_{ 5 70} \left( {\text{control}} \right) \times 100\%$$

where OD_570_ (sample) and OD_570_ (control) are the UV absorption of the sample and control groups at the wavelength 570 nm, respectively.

### Quantification of algal cells adhering to samples

The amount of algal cells adhering to sample was quantified from the chlorophylls released from the adherent algal cells. Briefly, non-adherent algal cells were removed following a gentle wash with PBS, pH = 7.4. The sample was then immersed in 3 ml DMSO, and placed on a rotary shaker with a speed of 150 rpm at 30 °C for 1 h. The extract was filtered through the 0.22 μm filter membrane (Tianjin Jinteng Experimental Equipment Co., Ltd, China), and the UV absorption was recorded using UV spectrophotometry at 428.5 nm.

## Results and discussion

### Chemical modification by MOI monomer

Silk fibers were chemically modified by MOI monomer in an anhydrous environment. Di-*n*-butyltin (IV) dilaurate were used as a catalyst effectively promoted the reaction between the isocyanate group and primary alcohol groups in silk fibers. The reaction was proceeded at 35 °C based on a previously published method [5]. We plotted the weight gain in SF fibers modified with MOI monomer against the reaction time at 35 °C. The mass-gain curve was sigmoid (the mass-gain curve is presented in Additional file 1: Figure S2). Weight gain was marked with increasing reaction time, finally plateauing at 20.6 wt% after a reaction time exceeding 24 h. Our reaction was therefore conducted at 35 °C for 24 h [5].

**Fig. 1:**
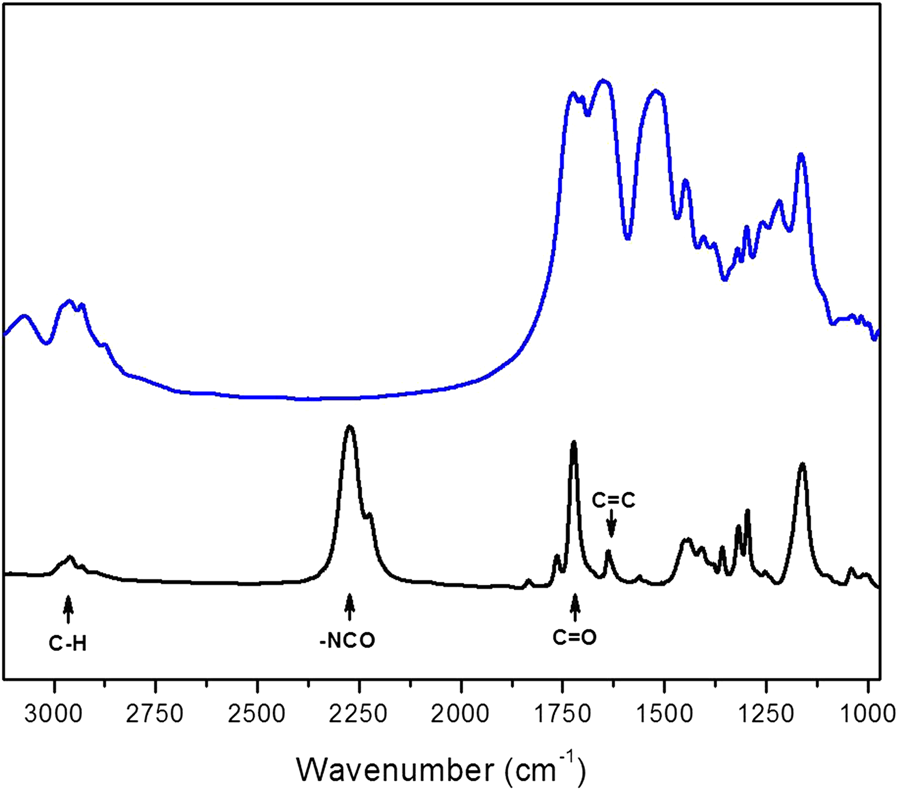
FTIR spectra of MOI monomer (black line) and MOI-modified silk (blue line)

FTIR spectra (Fig. 1) show that isocyanate and ester carbonyl peaks were clearly seen at 2270 and 1722 cm^−1^ in MOI monomer spectrum, respectively. The isocyanate peak in the MOI-modified silk spectrum completely disappeared after the reaction between SF and MOI, which indicates that unreactive MOI monomer did not remain on the MOI-modified silk. Thus, the chemical modification with MOI onto the silk fibers at 35 °C was successful, although IR absorption attributed to vinyl C=C stretching [12] could not be confirmed by FTIR analysis due to its overlapping with amide I of silk fibroin.

### Graft MOI-modified silk fibers with PFDT

PFDT was grafted at vinyl groups conjugated on the MOI-modified silk fibers using a thiol-ene click reaction. To determine the selectivity of the thiol-ene click reaction, we illuminated MOI-modified silk fibers with PFDT and FTOH molecules. We chose PFDT and FTOH molecules because the fluorine atom can serve as an elemental tag. In addition, as a highly hydrophobic molecule, it is expected that PFDT molecules can provide antifouling behavior against algal cells attachment [9]. Reaction progress was tracked with XPS measurements, and the F/C ratio was used to compare the number of fluorine-containing molecules attached to the surface of each sample.

**Fig. 2:**
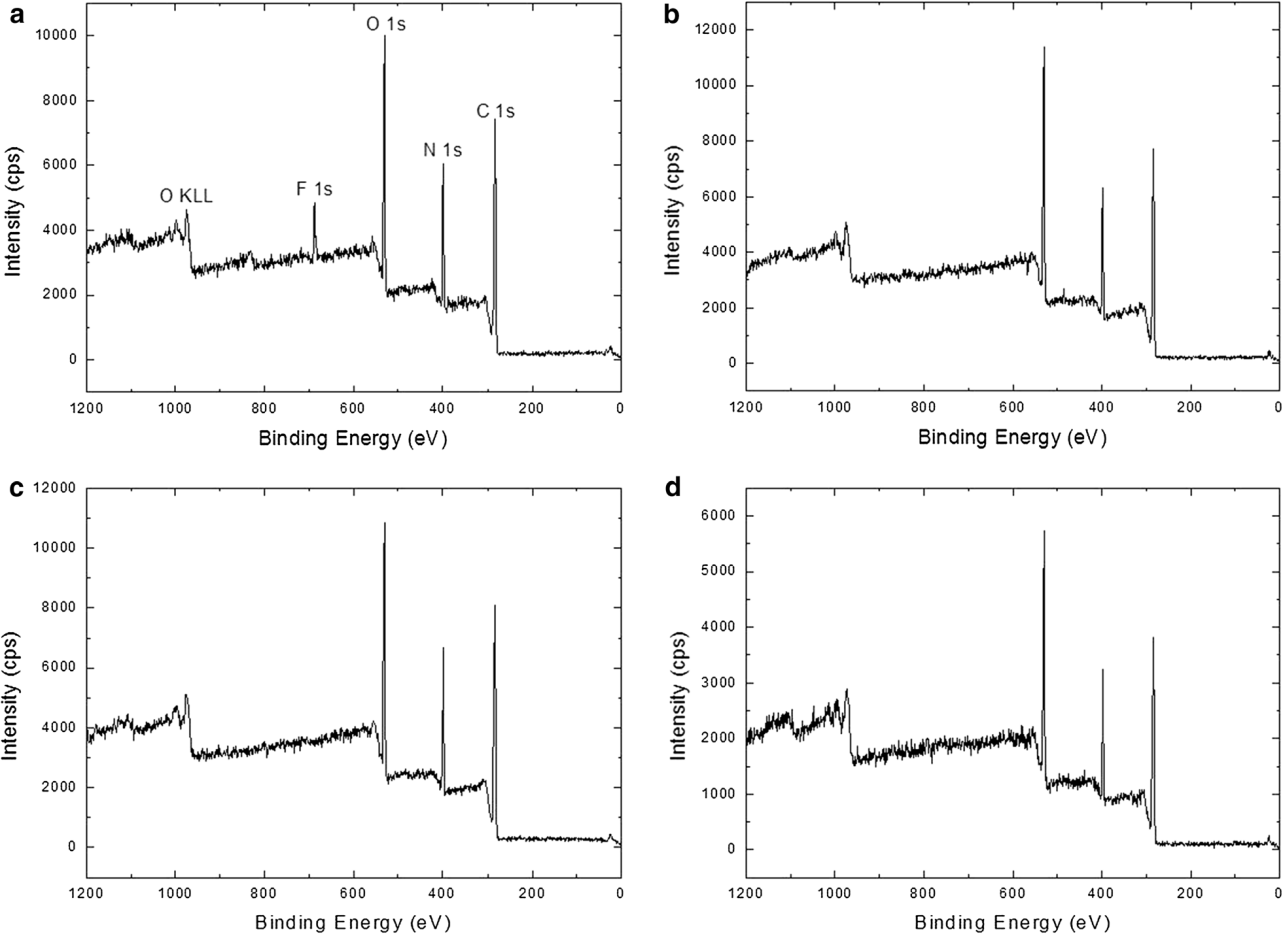
Representative XP survey spectrum of MOI-modified silk with neat PFDT under 365 nm UV illumination for 60 min (**a**), representative XP survey spectrum of MOI-modified silk with neat PTOH under 365 nm UV illumination for 60 min (**b**), representative XP survey spectrum of original silk with neat PFDT under 365 nm UV illumination for 60 min (**c**), and representative XP survey spectrum of MOI-modified silk with neat PFDT without UV illumination (**d**). Binding energy assignments are as follows: O KLL: 990 eV; F1s: 686 eV; O1s: 532 eV; N1s: 400 eV; C1s: 285 eV

**Scheme 1:**
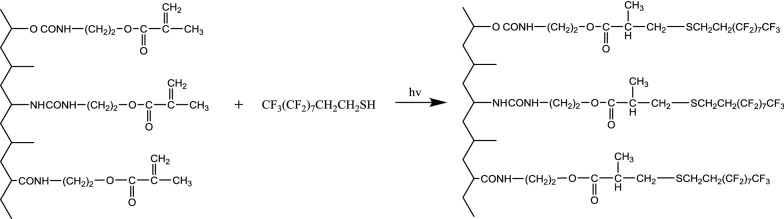
Preparation of PFDT-grafted silk via “thiol-ene” click reaction

When MOI-modified silk fibers were contacted with neat PFDT and illuminated under 365 nm UV light for 60 min, a fluorine peak could be detected by XPS (Fig. 2a) as a result of the thiol-ene click reaction between MOI-modified silk fibers and PFDT molecules (Scheme 1), with a fluorine-to-carbon (F/C) ratio of 0.03163 ± 0.00323. We also illuminated MOI-modified silk fibers in the presence of neat FTOH, the alcohol-containing analogue of PFDT, and we were unable to detect fluorine with XPS (Fig. 2b, F/C ratio = 0). Therefore, terminal thiol is necessary for the molecule attachment to the MOI-modified silk fibers.

To determine if PFDT attachment was specific to the MOI modification, we illuminated silk fibers without MOI modification in the presence of PFDT molecules. As a result, a 60 min illumination time resulted in no detectable fluorine electrons (Fig. 2c). The above observation supports the notion that an alkene group is necessary for the surface attachment through thiol-ene click chemistry.

Specifically, we incubated MOI-grafted silk fibers in the presence of PFDT molecules but in the absence of UV illumination before thoroughly rinsing and sonicating. A signal from fluorine electrons was not detectable by XPS (Fig. 2d), indicating that UV illumination of the silk fibers is required for the thiol-ene click reaction to proceed.

**Fig. 3:**
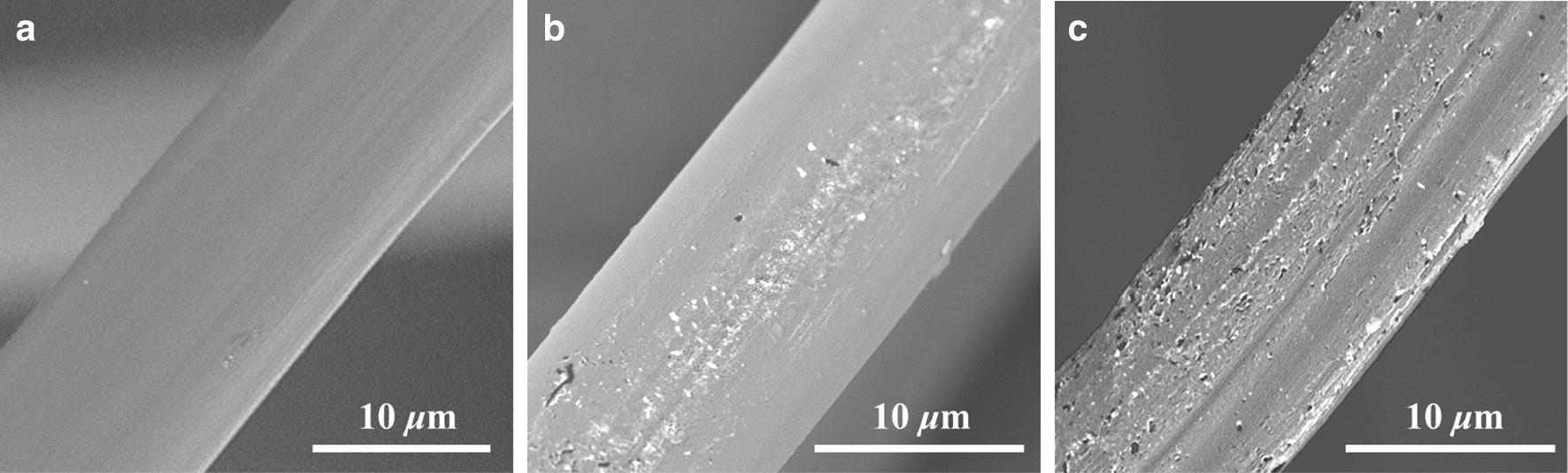
SEM photograph of the original **a** MOI-modified, **b** and PFDT grafted, **c** SF fibers

Figure 3 shows the SEM photograph of the original, MOI-modified, and PFDT grafted SF fibers. It seems that neither MOI nor PFDT are bonded to the SF fibers in layers. Instead, they are aggregated to form particles.

**Fig. 4:**
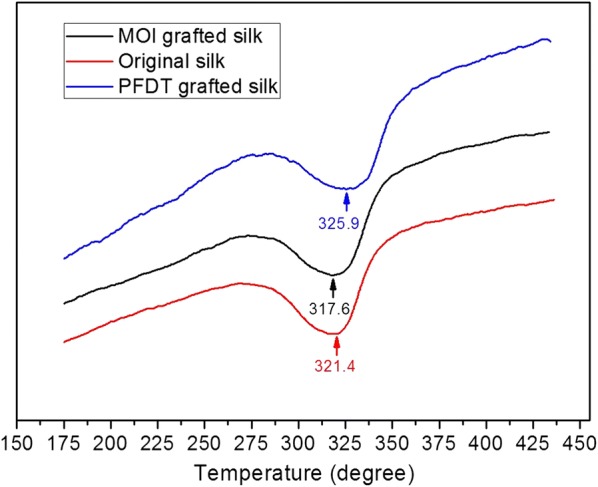
Representative DSC curves of original silk fibers, MOI-modified silk fibers, and PFDT-grafted silk fibers with UV light illumination for 60 min

We conducted DSC measurement to investigate the thermal behavior of the original silk fibers, the MOI-grafted silk fibers, and the PFDT-grafted silk fibers (Fig. 4). The MOI-grafted silk fibers have a thermal degradation temperature of 316.5 ± 1.0 °C (reported values are the average and standard deviation of n = 3 samples). This result indicates that the endothermic peaks of the MOI-grafted silk fibers were shifted to a lower temperature compared to that of the original silk fibers (320.6 ± 0.7 °C). As hydroxyl, carboxyl, and amino side groups of amino acid residues in silk fibroin would be replaced by MOI molecules, which are methyl and vinyl-terminated, it is estimated that the original intermolecular interactions-such as hydrogen bonds-would be disrupted, therefore resulting in decreased thermal stability of silk fibers. While the DSC curves of PFDT-grafted silk fibers have a thermal degradation temperature of 324.9 ± 0.9 °C, which shows a shift to higher values compared to the thermal degradation temperature of the original ones. As fluorine-containing molecules have superior thermal stability [13], this behavior can be explained by successful PFDT grafting onto the silk fibers. In summary, although thermal degradation temperatures of silk fibers shifted upon chemical modification at each step, the samples did not show any significant change in their thermal behaviors.

### Cell viability assay

**Fig. 5:**
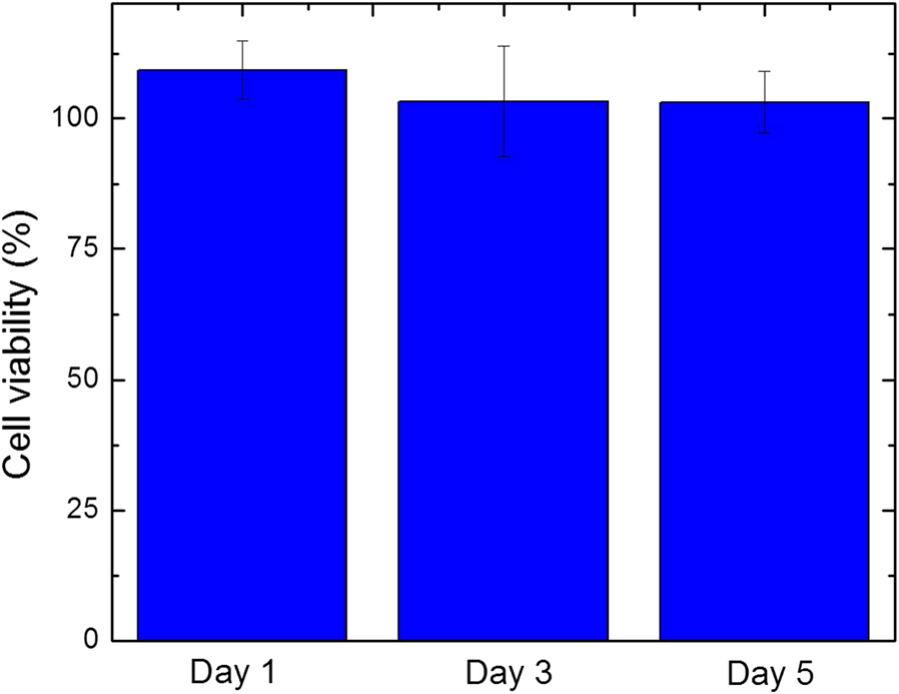
In vitro viability toward HEK 293 cells after incubation with leaching liquor from PFDT-grafted silk fibers for 24 h, 72 h, and 120 h respectively

To investigate the toxicity profile of the PFDT-grafted silk fibers, the standard MTT cytotoxicity assay [14] with Human Embryonic Kidney 293 (HEK 293) cells was performed. The cells were treated with leaching liquor from PFDT-grafted silk fibers. The cell viability of the samples were higher than that of control group, suggesting PFDT-grafted silk fiber exhibited high biocompatibility, which is the essential characteristic for biomaterials (Fig. 5).

### Algal cells adhesion experiment

To evaluate the antifouling performance of PFDT-grafted silk fibers and confirm the successful binding of PFDT on the surface of silk fibers via thiol-ene click chemistry, we immersed the PFDT-grafted silk fibers in a *C. vulgaris* culture for 1 week and 2 weeks, respectively. The cell-cultured samples were rinsed with a phosphate-buffered solution (PBS, pH = 7.4) three times to remove all non-adherent cells. The samples were then consecutively immersed in DMSO to extract chlorophyll, as described above. The amount of cells adhered to the samples was evaluated with UV spectrophotometry at a wavelength of 428.5 nm by measuring the chlorophyll content.

**Fig. 6:**
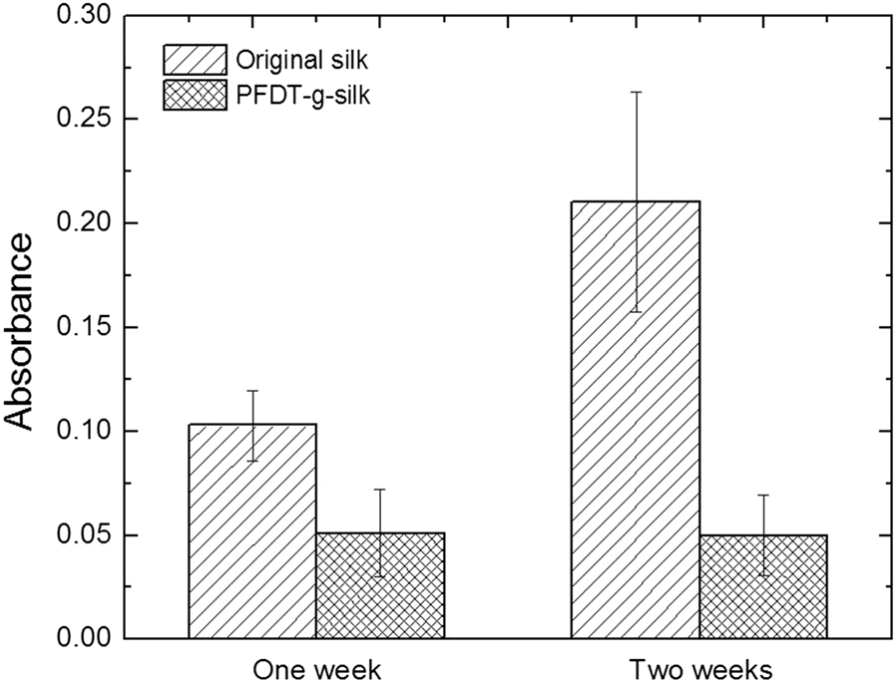
Comparison of *C. vulgaris* cell adhesion on the surface of original silk fibers and PFDT-grafted silk fibers (PFDT-g-silk) after 1 week and 2 weeks of incubation. Each bar represents the average and standard deviation of n = 3 samples

It was revealed that the PFDT-grafted silk fibers demonstrated less cell adhesion over both 1-week and 2-week algal cell culture periods (Fig. 6). In addition, although the amount of *C. vulgaris* cells adhered to original silk fibers increased as the increased incubation time, cells adhered to the PFDT-grafted silk fibers remain stable. Above results demonstrate good antifouling properties of the PFDT-grafted silk fibers and indicate the importance of surface chemical structure changes of silk fibers in preventing algal cell adhesion. PFDT incorporation changed algal cell adhesion because the hydrophobic moiety was introduced onto the surface of the silk fibers.

## Conclusion

In this work, we installed vinyl groups onto the silk fibroin fibers. Then, PFDT, as a model molecule, was grafted onto silk fibers using a thiol-ene click reaction in a heterogeneous system. Through different experimental designs, we specifically determined that (i) a thiol group of the functional molecule is required for attachment; (ii) vinyl groups on the silk fibers are needed for molecule attachment; and (iii) UV illumination of the sample is necessary for the reaction to proceed. *C. vulgaris* adhesion significantly decreased on PFDT-grafted silk fiber compared to original silk fibers. This result indicates that antifouling property can be lent to silk, and a thiol-ene click reaction initiated by UV illumination provides a method of grafting functional molecules onto silk fibers. This work could provide new perspectives for the application of the chemical modification techniques for silk fibroin and will also expand the spectrum of silk utilization. We believe that further reaction optimization (e.g. design a light source which could illuminate silk fibers with UV light evenly) would realize a controlled graft density of the functional molecules on the silk fibers, therefore facilitating the alternation of chemical and physical properties of silk fibers.

## Supplementary information

**Additional file 1: Figure S1.** Optical density of the *Chlorella vulgaris* culture at 660 nm as a function of time. **Figure S2.** Weight gain of MOI monomer on SF fibers as a function of reaction time. The weight gain of SF fibers after MOI modification was calculated as follows: weight gain (wt%) = 100 × (W_2_ − W_1_)/W_1_, where W_1_ and W_2_ are the dried original SF fibers and MOI-modified SF fibers, respectively.

## Data Availability

The datasets used and analysed during the current study are available from the corresponding author on reasonable request.

## Abbreviations

SF: silk fibroin
MOI: 2-methacryloyloxyethyl isocyanate
PFDT: 1*H*,1*H*,2*H*,2*H*-perfluorodecanethiol
FTOH: 1*H*,1*H*,2*H*,2*H*-perfluoro-1-decanol
XPS: X-ray photoelectron spectroscopies
FTIR: Fourier transform infrared
SEM: scanning electron microscopy
DSC: differential scanning calorimeter
DMF: *N*,*N*-dimethylformamide
DMSO: anhydrous dimethyl sulfoxide
EDC: 1-ethyl-3-(dimethylaminopropyl) carbodiimide hydrochloride
NHS: *N*-hydroxysuccinimide
*C. vulgaris*: *Chlorella vulgaris*
LED: light-emitting diode
