# Supplementary material for: Chemical modification of Bombyx mori silk fibers with vinyl groups for thiol-ene click chemistry
Source: BMC Chem. 2019 Sep 10;13(1):114. doi: 10.1186/s13065-019-0630-7 (PMC6734490; doi:10.1186/s13065-019-0630-7)
Supplement: Supplementary file 1 — Additional file 1: Figure S1. Optical density of the Chlorella vulgaris culture at 660 nm as a function of time. Figure S2. Weight gain of MOI monomer on SF fibers as a function of reaction time. The weight gain of SF fibers after MOI modification was calculated as follows: weight gain (wt%) = 100 × (W2 − W1)/W1, where W1 and W2 are the dried original SF fibers and MOI-modified SF fibers, respectively. [file 13065_2019_630_MOESM1_ESM.docx]

**Additional file**

Figure S1. Optical density of the *Chlorella vulgaris* culture at 660 nm as a function of time.

Figure S2. Weight gain of MOI monomer on SF fibers as a function of reaction time. The weight gain of SF fibers after MOI modification was calculated as follows: weight gain (wt%) = 100 × (W_2_-W_1_)/W_1_, where W_1_ and W_2_ are the dried original SF fibers and MOI-modified SF fibers, respectively.
